# Reporting preclinical anesthesia study (REPEAT): Evaluating the quality of reporting in the preclinical anesthesiology literature

**DOI:** 10.1371/journal.pone.0215221

**Published:** 2019-05-23

**Authors:** Dean A. Fergusson, Marc T. Avey, Carly C. Barron, Mathew Bocock, Kristen E. Biefer, Sylvain Boet, Stephane L. Bourque, Isidora Conic, Kai Chen, Yuan Yi. Dong, Grace M. Fox, Ronald B. George, Neil M. Goldenberg, Ferrante S. Gragasin, Prathiba Harsha, Patrick J. Hong, Tyler E. James, Sarah M. Larrigan, Jenna L. MacNeil, Courtney A. Manuel, Sarah Maximos, David Mazer, Rohan Mittal, Ryan McGinn, Long H. Nguyen, Abhilasha Patel, Philippe Richebé, Tarit K. Saha, Benjamin E. Steinberg, Sonja D. Sampson, Duncan J. Stewart, Summer Syed, Kimberly Vella, Neil L. Wesch, Manoj M. Lalu, on behalf of the Canadian Perioperative Anesthesia Clinical Trials Group

**Affiliations:** 1 Clinical Epidemiology Program, Blueprint Translational Research Group, The Ottawa Hospital Research Institute, Ottawa, Ontario, Canada; 2 Faculty of Medicine, University of Ottawa, Ottawa, Ontario, Canada; 3 Department of Anesthesia, McMaster University, Hamilton, Ontario, Canada; 4 Department of Medicine, McMaster University, Hamilton, Ontario, Canada; 5 Department of Anesthesiology and Pain Medicine, University of Alberta, Edmonton, Alberta, Canada; 6 Department of Anesthesiology and Pain Medicine, The Ottawa Hospital, Ottawa, Ontario, Canada; 7 Department of Innovation in Medical Education, University of Ottawa, Ottawa, Ontario, Canada; 8 Department of Anesthesiology and Perioperative Medicine, Queen’s University, Kingston, Ontario, Canada; 9 Department of Anesthesia, Pain Management & Perioperative Medicine, Dalhousie University, Halifax, Nova Scotia, Canada; 10 Department of Anesthesia, University of Toronto, Toronto, Ontario, Canada; 11 Discipline of Anesthesia, Memorial University, St. John’s, Newfoundland and Labrador, Canada; 12 Department of Anesthesiology, Université de Montréal, Montréal, Québec, Canada; 13 Regenerative Medicine Program, The Ottawa Hospital Research Institute, Ottawa, Ontario, Canada; 14 Department of Cellular and Molecular Medicine, University of Ottawa, Ottawa, Ontario, Canada; Johns Hopkins University Bloomberg School of Public Health, UNITED STATES

## Abstract

Poor reporting quality may contribute to irreproducibility of results and failed ‘bench-to-bedside’ translation. Consequently, guidelines have been developed to improve the complete and transparent reporting of *in vivo* preclinical studies. To examine the impact of such guidelines on core methodological and analytical reporting items in the preclinical anesthesiology literature, we sampled a cohort of studies. Preclinical *in vivo* studies published in *Anesthesiology*, *Anesthesia & Analgesia*, *Anaesthesia*, and the *British Journal of Anaesthesia* (2008–2009, 2014–2016) were identified. Data was extracted independently and in duplicate. Reporting completeness was assessed using the National Institutes of Health Principles and Guidelines for Reporting Preclinical Research. Risk ratios were used for comparative analyses. Of 7615 screened articles, 604 met our inclusion criteria and included experiments reporting on 52 490 animals. The most common topic of investigation was pain and analgesia (30%), rodents were most frequently used (77%), and studies were most commonly conducted in the United States (36%). Use of preclinical reporting guidelines was listed in 10% of applicable articles. A minority of studies fully reported on replicates (0.3%), randomization (10%), blinding (12%), sample-size estimation (3%), and inclusion/exclusion criteria (5%). Statistics were well reported (81%). Comparative analysis demonstrated few differences in reporting rigor between journals, including those that endorsed reporting guidelines. Principal items of study design were infrequently reported, with few differences between journals. Methods to improve implementation and adherence to community-based reporting guidelines may be necessary to increase transparent and consistent reporting in the preclinical anesthesiology literature.

## Introduction

The successful translation of preclinical research to the clinical setting often depends on promising results or signals from animal experiments. In practice, approximately 27% high impact preclinical findings lead to in-human trials and only around 5% are translated [1–3] from ‘bench-to-bedside’ (approved for clinical use). It has been suggested that this poor level of translation may be due to a lack of reproducibility within basic science studies [4, 5]. Widely cited factors influencing this irreproducibility are poor preclinical study design, incomplete reporting, and a lack of transparency of results [4, 6, 7]. Reporting of critical elements such as randomization, blinding, and sample size estimation allow for comparison between experiments and assessment of internal validity. Poorly designed, executed, and reported preclinical studies have contributed to the replication crisis, and increase waste of research funding, laboratory animals, and personnel time [8, 9]. Despite this recognition, previous reviews of experimental animal research found key elements, such as randomization and blinding, are infrequently reported [10, 11].

In response to poor reporting observed in preclinical studies, several stakeholders have developed reporting guidelines. The endorsement of similar efforts for the reporting of clinical trials and systematic reviews have led to significant improvements in the completeness of reporting for these types of studies [12, 13]. The ‘Animal Research: Reporting of In Vivo Experiments’ (ARRIVE) guidelines published in 2010 provided the first widely endorsed reporting standards for preclinical research [14]. In parallel, the National Institutes of Health (NIH) convened a number of stakeholders (scientists, funders, regulators, and journal editors) to provide a consensus on essential reporting items [4] in all preclinical experimental animal research, which was subsequently developed into the NIH preclinical reporting guidelines (NIH-PRG) [15]. The NIH-PRG selected a minimum core set of seven reporting domains from the ARRIVE guidelines that are included in any preclinical publication [15].

While some anesthesiology journals have endorsed preclinical reporting guidelines, and expert opinion has emphasized the importance of transparent reporting [16–19], the current level of reporting rigor in the preclinical anesthesiology literature against these core reporting domains is unknown. It is important to understand preclinical anesthesiology reporting for several reasons. First, since preclinical studies in anesthesiology encompass a variety of subject matter (e.g. cardiovascular, respiratory, pain, neuroscience, critical care), an assessment of this literature provides insights across biomedical research and are likely generalizable to the wider preclinical research community. Second, the high potential for ‘bench-to-bedside’ translation of work published in anesthesia journals–due to the publication of both preclinical and clinical results across numerous biomedical fields–also provides an added impetus to carefully assess aspects that reflect validity of findings [20]. Third, by providing a complete assessment of reporting future interventions can be tailored to the gaps identified. In addition, the current “epidemiology” of the preclinical literature has not been investigated (e.g. what topics are being investigated, what types of animals are being used, which countries contribute to this literature). In order to identify and address these evidence and knowledge gaps, we performed a cohort study of preclinical *in vivo* animal studies to appraise the quality of reporting and produce an evidence map of the current preclinical literature.

## Methods

### Protocol

Prior to study selection and data extraction, our protocol was deposited on the Open Science Framework (OSF) [21] and the University of Ottawa’s Open Access Research Institutional Repository [22]. The protocol was endorsed by the Canadian Perioperative Anesthesia Clinical Trials Group. Although this study is not a systematic review *per se*, the Preferred Reporting Items for Systematic reviews and Meta-Analyses Protocol (PRISMA-P) [23] and PRISMA [24] were used as general guidelines in reporting the protocol and this manuscript, respectively.

#### Primary review objective and outcomes

As outlined in our protocol [21], the focused research question we addressed was: how completely do *in vivo* preclinical studies in anesthesiology journals adhere to core reporting standards for rigorous study design? Our primary outcome was: completeness of reporting as assessed by the core set of reporting standards suggested by the NIH. An exploratory analysis was also planned *a priori* [21] to compare reporting over time and between journals.

#### Eligibility criteria

We included articles of original research using *in vivo* animal models published in *Anesthesiology*, *Anesthesia & Analgesia (A&A)*, *Anaesthesia*, and the *British Journal of Anaesthesia (BJA)*. These four journals were selected as they had the highest impact factor of all general anesthesiology journals in Thomson Reuters’ Journal Citation Reports in 2016, and they include investigations on a wide number of domains of biomedical science. Articles from the journal *Pain* were not considered as its scope is significantly limited compared to the included general anesthesiology journals. There were no limitations on the disease model, intervention, comparisons, outcomes, or experimental design. Any *in vitro*, *ex vivo*, or clinical studies were excluded. Abstracts, letters, reviews, and commentaries were also excluded. The first widely endorsed preclinical reporting guidelines, ARRIVE [14], were published in 2010 and they were endorsed by *Anaesthesia* and the *BJA*. Thus, in order to assess potential uptake in reporting practices over time in our exploratory analysis we selected articles published in 2014–2016 to account for potential time in implementation of these first endorsed reporting guidelines throughout the preclinical research community. As a comparator, articles published in 2008–2009 were chosen (i.e. prior to the publication of the first endorsed preclinical reporting guidelines). Articles from 2010–2013 were not included. As noted above, the ARRIVE guidelines include all elements found in the NIH-PRG, however they are more expansive and include many elements not deemed essential by the NIH-PRG.

#### Search strategy and article screening

A search was developed and conducted by an information specialist to identify all eligible articles through MEDLINE, which indexes all four journals. Two independent reviewers performed the process of study selection. Title assessment used a liberal accelerated screening method (one reviewer required to process, two required to exclude) [25]. Each study was screened by its abstract and then full-text by two independent reviewers (two reviewers required to process, two reviewers required to exclude). Journal classification of articles (e.g. basic or clinical science) was not considered when selecting studies for inclusion (i.e. only our pre-specified eligibility criteria were used for study selection). Consensus was required to include a study and any conflicts were resolved through consultation with the senior author.

#### Data extraction

Those articles that met our inclusion criteria were retrieved and imported to audit-ready cloud-based software (Distiller SR, Evidence Partners; Ottawa, Canada). Due to the large number of studies that met the eligibility criteria, targeted crowdsourcing for data extraction and assessment was used (i.e. extractors were recruited through the Canadian Perioperative Anesthesia Clinical Trials Group). Extraction forms were pilot-tested and then uploaded to Distiller SR. To ensure an adequate level of reviewer agreement, all extractors participated in a calibration exercise. Each extractor reviewed a training document [21] and then independently extracted four articles not included in our sample that had been evaluated by the core study group. Extractors received individualized feedback via email and/or videoconference by one of the core group members. This process was then repeated with another four articles and training was deemed complete when the extractor achieved a high level of inter-rater agreement with the core study group’s assessments (inter-rater agreement greater than 80%). All extractors achieved this level of agreement within eight training articles. All articles were then assessed and data extracted in duplicate by independent reviewers. Information was extracted regarding the general characteristics of the study (e.g. country of residence of corresponding author, source of funding). Each study was also classified according to its broad topic of investigation using an algorithm based on topics identified by the International Statistical Classification of Diseases and Related Health Problems (ICD-10). Quality of reporting was assessed using the scheme described below. Extractors were not blinded to journal or date of publication. Conflicts between extractors were resolved by a core group member.

#### Reporting quality assessment of included studies

**Fig 1:**
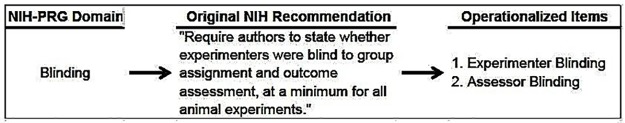
Constructing our reporting checklist. The National Institutes of Health preclinical reporting guidelines (NIH-PRG) consist of seven domains, each containing a multi-faceted recommendation. This recommendation for the domain of blinding was deconstructed and two unidimensional items were identified.

The NIH-PRG consist of a core set of seven domains: 1) use of community-based reporting standards, 2) distinguishing between biological and technical replicates, 3) statistics, 4) randomization, 5) blinding, 6) sample size estimation, and 7) inclusion/exclusion criteria. Since each domain encompasses complex, multifaceted concepts we operationalized each through deconstruction into 21 unidimensional items. Each item was then phrased as a simple ‘yes’ or ‘no’ question (S1 Table). For example, the domain of blinding was deconstructed into questions regarding both experimenters’ blinding (addressing performance bias) and blinding of assessments (addressing detection bias) (Fig 1). This 21 item checklist served as our reporting assessment tool. We note that one question regarding experimenter assessment blinding did allow for a ‘sometimes’ response to distinguish between blinding for some outcomes that are commonly assessed in a blinded manner (e.g. histology) versus other outcomes that are usually not (e.g. statistical analysis); this was pooled with the ‘yes’ responses for that question.

#### Statistical analysis

Descriptive statistics (i.e. frequency counts) were generated for each of the NIH-PRG items. The total number of times each item was reported (n) across all studies (N) was expressed both nominally (n of N) and as a percentage (n/N). Several exploratory comparative analyses were planned *a priori*. Changes in reporting between the two time periods (pre/post ARRIVE) were assessed by comparing reporting in studies published before and after 2010. Differences between reporting in journals were assessed between those that were early adopters in endorsing preclinical reporting guidelines (*BJA* and *Anaesthesia*) versus those that were not (*Anesthesiology* and *A&A*). Formal comparisons of proportions using risk ratios (RR) and 95% confidence intervals (CI) were performed using Comprehensive Meta-Analysis (Version 3, Biostat Inc.; New Jersey, U.S.A).

#### Deviations from protocol

A questions assessing reporting of replicates was found to have low inter-rater agreement and was replaced midway through the study with new questions (detailed in codebook posted on OSF [21]). Responses to the original question were not considered and the new questions were answered for all included studies. We did not find it necessary to perform normality testing on data. The sample size was large enough (N = 604) such that we felt comfortable assuming a normal distribution of data points. Furthermore, given the low rates of complete reporting, we did not believe it would be meaningful to perform an analysis per journal, per year, per item.

## Results

### Study characteristics

**Fig 2:**
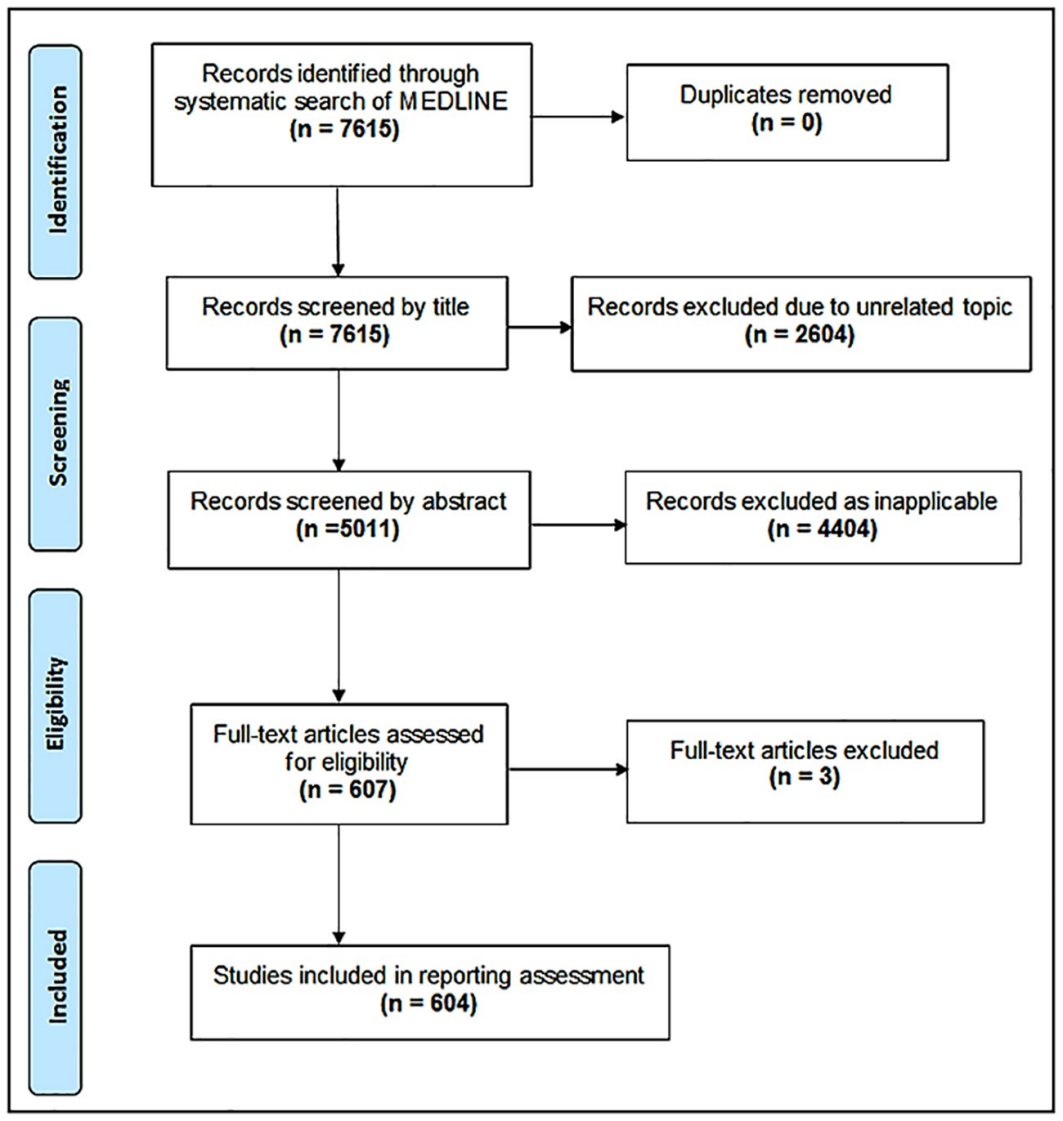
Preferred reporting items for systematic reviews and meta-analyses (PRISMA [24]) study selection diagram.

Our search identified 7615 records (Fig 2). Initial title and abstract screening excluded 7008 records with an additional 3 articles excluded following full-text review. In total, 604 articles were included for assessment in this study (a full list and our search strategy can be found on OSF [21]).

### Epidemiology of preclinical anesthesiology studies

**Fig 3:**
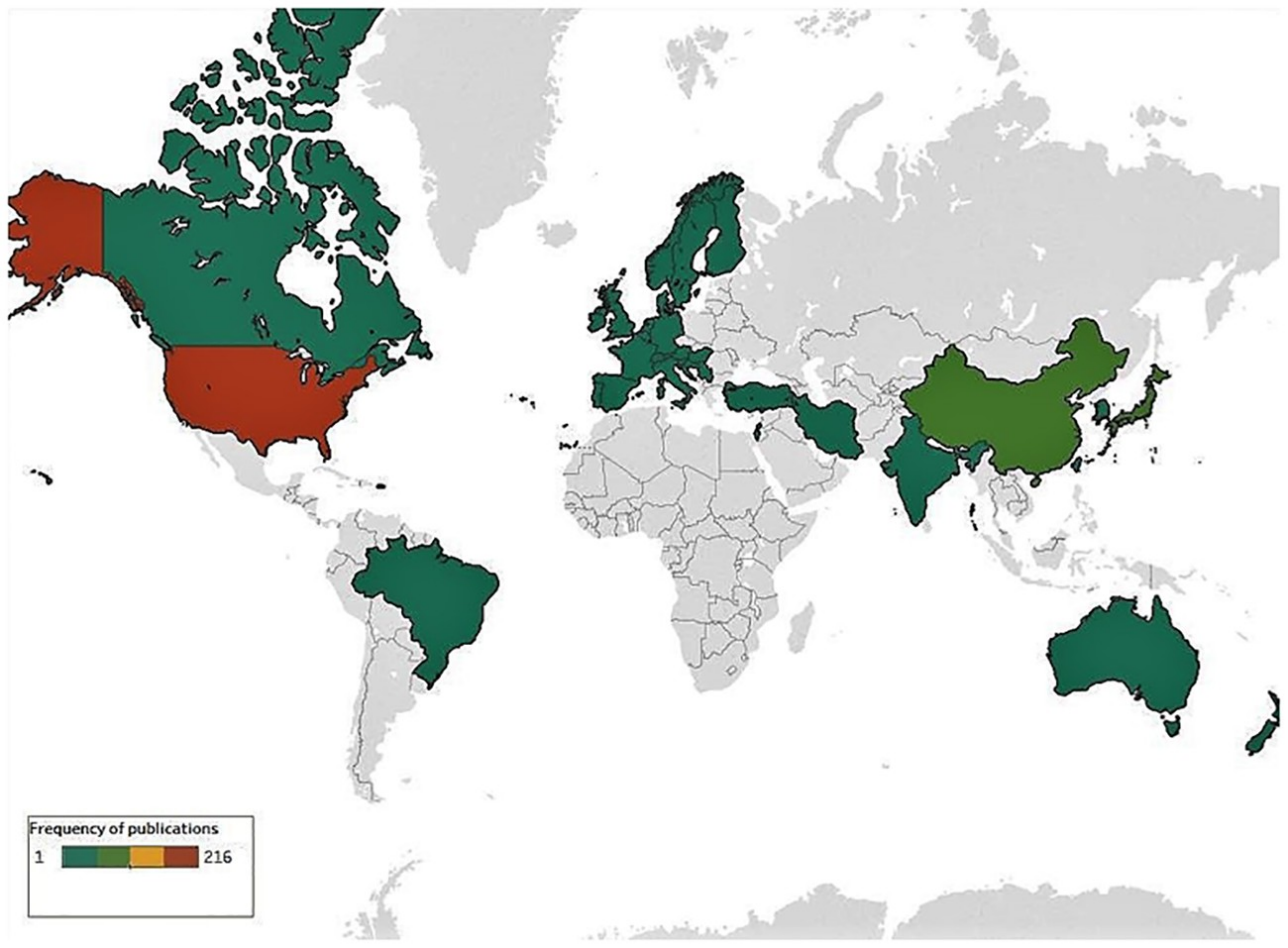
Distribution of publications. World map depicting the number of articles published per country based on the corresponding author’s residency at the time of publication (image created using Tableau Software; Seattle, Washington, United States).

Two hundred ninety-two articles (48%) were published in *Anesthesiology*, 235 (39%) in *A&A*, 70 (12%) in *BJA*, and 7 (1%) in *Anaesthesia*. The country of the corresponding author ranged across 32 countries (Fig 3 and S2 Table). Most common was the United States (n = 216, 36%), China (n = 66, 11%), Japan (n = 62, 10%), and Germany (n = 53, 9%). The three most frequently cited sources of research funding acknowledged by papers (out of a total of 891, due to multiple sources of funding) included government agencies (n = 408, 46%), academic institutions (n = 276, 31%), and private industry (n = 75, 8%) (S3 Table). Nineteen different broad topics of investigation were identified. Pain and analgesia was the most common topic (n = 180, 30%), while critical illness (n = 77, 13%), the cardiovascular system (n = 75, 12%), and the nervous system (n = 70, n = 12%) were also frequently the focus of studies (S4 Table). Animal models included 12 different species with a total of 617 different animal models used, but the majority of studies used rats (n = 338, 55%) and mice (n = 132, 21%) (S5 Table). A total of 52 490 animals were used in all experiments, including 32 223 rats and 8 983 mice. Three hundred seventy-seven articles (62%) had titles which clearly identified the work as preclinical research.

### Reporting characteristics related to bias

**Fig 4:**
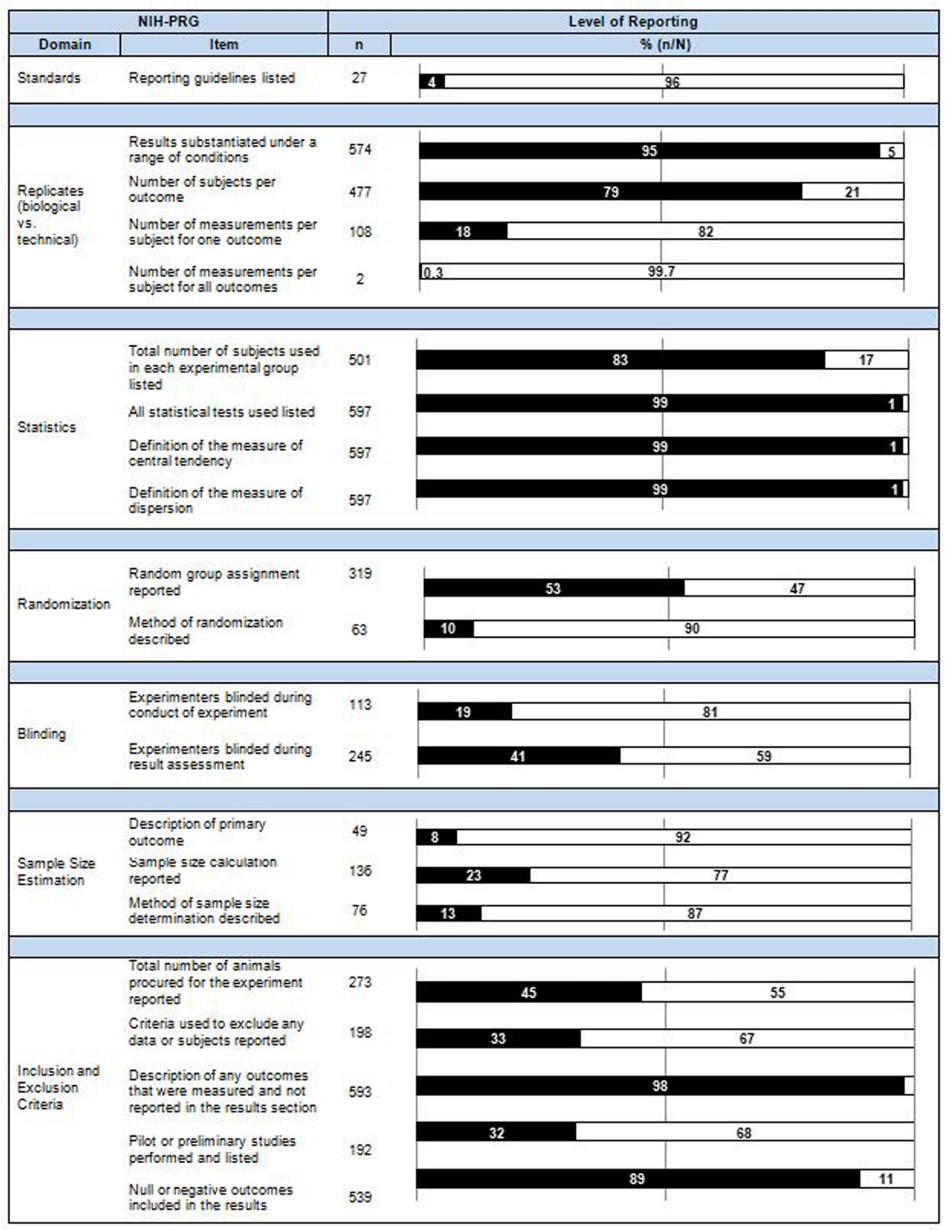
Reporting assessment results. Completeness of reporting across all included studies (N = 604) against the deconstructed NIH-PRG. The data is displayed by item in each domain as a frequency (n), and as a percentage (n/N), where black and white correspond to an item being reported or not reported, respectively.

Reporting in each of the seven domains outlined by the NIH-PRG was assessed. Within each section below a summary of guidance for each domain is provided to orient the reader to requirements suggested by the NIH-PRG. The collective results are displayed in Fig 4 and S6 Table. For items contained in our checklist inter-rater agreement was 86% during extraction (i.e. before consensus). The complete data set for each study can be found on OSF [21].

#### The use of reporting guidelines

The NIH-PRG encourage authors to use community-based nomenclature and reporting standards [15]. Twenty-seven of the 282 articles from after publication of the ARRIVE guidelines listed the use of reporting guidelines during study design and preparation of a manuscript for publication. Twenty-six of these articles cited the ARRIVE guidelines and the other listed Utstein-Style [26] guidelines for laboratory CPR research.

#### Measurement techniques

The NIH-PRG state that sufficient information should be provided to distinguish between technical and biological replicates; particularly the number of subjects used and measurements performed [15]. The number of subjects and measurements for at least one experimental outcome were reported in 477 (79%) and 108 (18%) articles, respectively. Both items were reported in 2 articles (0.3%). To increase potential external validity, it is required that investigators report whether the results were substantiated under a range of conditions [15]. Five hundred and seventy-four articles (95%) included results of studies over a range of conditions (e.g. different intervention dosages or durations). All items relating to replicates were completely reported in 2 articles (0.3%).

#### Statistics and sample size

Full reporting of statistics requires authors state the statistical tests used and exact value of n, as well as define the center and measure of dispersion [15]. The statistical tests used were reported in 597 articles (99%). The number of animals per group was reported in 501 articles (83%), either as explicit sample sizes or more commonly as ranges. In 597 studies (99%) the measures of central tendency and dispersion were both stated. Overall, 492 articles (81%) reported on all items relating to statistics.

Reporting of an *a priori* sample size calculation to determine an appropriate number of subjects and a description of the power calculation is contained in the recommendations [15]. Calculation of a sample size is typically dependent on declaration of a primary outcome, which was explicitly stated in 49 articles (8%). In 136 studies (22%) the use of a sample size calculation was reported. Of these studies, the statistical method or a rationale for the sample size was described in 76 articles (56%). In total, 18 articles (3%) described all elements required to adequately justify sample size (i.e. all the following were reported: primary outcome stated, sample size declared, and rationale for sample size provided).

#### Randomization and blinding

For applicable experiments, the randomization of animals and the method of randomization must be stated, as these reduce selection bias [15]. In 574 studies (95%) there were experimental designs that included multiple arms, which may signal the ability to randomize. Of these, 319 (56%) reported on random group assignment. Of the 319 articles that randomized animals into experimental groups, 63 (10%) stated the specific method of randomization. Across all studies 10% reported both randomization and method of randomization.

The NIH-PRG recommend authors report whether experimenters were blinded to group assignment (to reduce performance bias) and outcome assessment (to minimize detection bias) [15]. The blinding of personnel conducting experiments was described in 113 studies (19%). Personnel assessing outcomes were reported as being blinded to all outcome assessments in 57 articles (9%). In 188 articles (31%) personnel were blinded to some outcome assessments, most frequently assessment of histology (55% of these studies). Blinding of experimenters performing the study and assessing (some or all) outcomes were both reported in 72 articles (12%).

#### Inclusion and exclusion criteria

In order to minimize selection bias, the NIH-PRG require the criteria for exclusion of any subjects or results to be clearly stated [15]. In order to understand the flow of animals through an experiment and potential exclusions, the total number of animals must first be transparently reported; this was stated in 273 articles (45%). Exclusion of any data, or lack thereof, was reported in 198 articles (33%).

In clinical studies, selective outcome reporting can be detected by comparing registered protocols with final reports; as preclinical studies are not routinely registered *a priori*, we instead compared experimental design as described in the methods section to the reported results. We found 593 articles (98%) reported the results from all experiments described in the methods section.

In order to increase transparency of the development of preclinical study design, the NIH-PRG suggest that any pilot or preliminary experiments, especially those that do not support the main findings (null or negative results) be reported [15]. Pilot or preliminary results were reported in 192 studies (32%). In our sample of studies, null or negative results were stated in 539 studies (89%) most commonly through negative results within a range of conditions (e.g. a dose-response curve with doses that do not produce the measured outcome). Reporting of all items recommended in the inclusion/exclusion criteria domain was found in 32 articles (5%).

#### The effect of reporting standards—Exploratory analysis

Twenty-seven articles (4% of the total) stated that they used reporting guidelines when designing their study and preparing a manuscript summarizing their results. Completeness of reporting was compared between articles that listed reporting guidelines (N = 27, 5%) and articles that did not (N = 577, 96%) (S7 Table). We found completeness of reporting did not meaningfully alter any of the NIH-PRG items.

#### Reporting practices between journals—Exploratory analysis

A comparison of completeness of reporting for each NIH-PRG item was also performed for articles published in journals which endorsed the preclinical reporting guidelines (*BJA* and *Anaesthesia*) and journals which had not (*Anesthesiology* and *A&A*) (S8 Table). The journals that did not endorse guidelines included 241 articles and the group that did endorse guidelines included 41 articles. We found that endorsement led to a meaningful increase in the listing of reporting guidelines (59% compared to 0.8%; RR 70.54, 95% CI 17.33–287.18). Again, we found meaningful increases in key items such as the total number of animals procured, but also found notable decreases, such as for reporting the method of random group assignment. Most items showed no statistically significant change.

#### Reporting practices over time—Exploratory analysis

Articles published in 2008 and 2009 (prior to the first endorsed reporting guidelines; N = 322, 53%) was compared to those from 2014–2016 (N = 282, 47%) (S9 Table). Increases in the level of reporting were noted for important items, such as sample size estimation, but decreases were also present for items such as reporting the total number of animals used. The majority of items showed no statistically significant difference.

## Discussion

This review provides the most comprehensive assessment of the epidemiology and reporting quality in the preclinical anesthesiology literature to date. Our reporting assessment demonstrates that basic components of experimental design and key elements in study methodology, such as blinding, randomization, and sample size estimation, are suboptimally reported.

*In vivo* preclinical experiments offer physiological insights into clinical conditions as well as justification for early phase clinical trials. Thus, it is imperative that readers are able to appraise the validity of outcomes and conclusions in preclinical studies. To better evaluate and improve animal experiments, preclinical studies should incorporate the same elements as clinical studies. For instance, it is well known that rigorous study design reduces bias in the clinical setting [27–30]. Not surprisingly, the absence of basic elements such as randomization and blinding has been associated with biased (exaggerated) effect sizes in preclinical studies [31–33]. The majority of studies in our sample failed to indicate whether selection bias was addressed, through randomization and the particular method used. Failing to report the method of randomization is particularly problematic as it remains unclear whether the particular technique used was optimal (e.g. true randomization such as a computer package) or suboptimal (e.g. pseudo-randomization methods such as ‘picking cages at random’).

Similarly, blinding of group allocation was absent in the majority of studies (which increases risk of selection and information bias) and blinding of outcome assessment was not performed for most outcomes. Interestingly, blinding was reported frequently for histology, which reflects the standard in basic science for ascertainment of this outcome to be performed in a blinded manner. It is unclear why blinding is not more widely adopted for other outcomes at the bench, but this may reflect a lack of awareness of the importance of addressing key items of internal validity in preclinical experimental design [34]. One can speculate that lack of blinding can result, in part, from lack of resources or personnel, since typical practice in basic science laboratories is such that a single graduate student or research associate is involved in both data acquisition and data analyses for a given experiment or project.

One of the aims of the NIH-PRG is to optimize the use of animals in preclinical experiments. Over 50 000 animals were used, yet despite this immense use of resources and animal lives, sample size calculations were reported in only a third of articles. This suggests that experimenters either based sample sizes on previous experience or did not consider the need for formal calculations (or alternatively did not report it) [35]. This leads to a potential waste of resources as studies may use an unwarranted number of animals; conversely, a lack of sample size calculations may lead to under-powering of preclinical studies which undermines the strength of their findings [36]. Another issue related to animal use is the failure to report the exact number of animals entering a study along with the number that were analyzed for each outcome (less than a fifth of studies reported the precise number of subjects used for all experiments). Collectively, this suggests that animal use is likely underreported and that their use may not be optimized. The ethics and scientific integrity of preclinical studies is undermined by this lack of accounting of animals.

The failure to use good methodological practices increases risk of bias and may also speak to a lack of understanding of these methods. Perhaps most telling were articles stating they adhered to reporting guidelines, but rarely reported required elements. This latter result demonstrates misunderstanding and/or misinterpretation of domains listed by reporting guidelines. It also reflects the fact that endorsement of reporting guidelines does not automatically entail enforcement. It may also demonstrate that further education around these issues is required for scientists who perform *in vivo* bench research.

### Study limitations

Several limitations to our study should be considered. It is possible that authors did employ methods that were evaluated (e.g. randomization), however failed to report them. Thus, our reported rates of complete reporting would under-represent the actual use of these methods. Second, only the top four journals by impact factor in the anesthesiology literature were included in our review. We selected journals based on impact factor as they publish articles that are deemed to be of high priority. Nonetheless, it is unclear what completeness of reporting may have been in a random sample of journals. Previous studies have found either no relationship [37, 38] or a negative association [39] between completeness of reporting and impact factor. Third, we note that selective outcome reporting is inherently difficult to assess in preclinical studies as publications often only highlight positive results obtained after study completion, rather than all outcomes investigated. No studies contained in our assessment registered a study protocol *a priori*, thus we were limited to comparing methods and results sections of each publication. Last, our *a priori* planned comparative analyses should be considered exploratory. Future studies with larger sample sizes may be able to more robustly assess factors in the comparative analysis.

### Future steps

We believe a number of potential solutions exist that may improve reporting in the preclinical anesthesia community. First, we believe that further education of basic scientists on study design and key items to preserve internal validity evaluation is needed [40]. Both journals and anesthesia research societies should take steps to promote and disseminate methods to reduce bias at the bench. At an institutional and departmental level, more can be done to integrate biostatisticians and methodologists into preclinical research, similar to the current integration they now have with clinical research. At the scientist and trainee level, specific barriers to the implementation of the methods described in reporting guidelines need to be assessed and addressed [41]. Authors should not fear transparent reporting, as implementation of methods described may not always be feasible (e.g. blinding may not be possible in a study conducted by one graduate student at the bench). In these cases, transparent reporting will allow readers to evaluate potential risk of bias. In addition, support for rigorous study design by funding agencies may provide further impetus for change [42]. This is particularly important at a time when the benefit of animal research to humans is being questioned [43, 44]. Measures that could also be considered by journals are mandatory checklists on submission of a manuscript [45], along with training reviewers to understand the 7 recommended domains. Last, we mention that preregistration of study protocols (e.g. www.preclinicaltrials.eu)–common practice in clinical research–would allow readers to assess whether the final publication reflected the original and intended outcomes. Ultimately, improving replication at the bench, and potentially increasing successful clinical implementation of preclinical research, is dependent upon rigorous study design and transparent reporting of conduct.

### Conclusions

This is the first assessment of a body of preclinical research against all items of the NIH-PRG. It is evident that endorsement of preclinical reporting guidelines has not led to substantive changes in quality/completeness of reporting. Future investigations will help delineate whether uptake increases and is retained. Clearly further efforts will be needed to promote a paradigm shift in the culture of transparency and complete reporting in preclinical studies. Efforts by journals to endorse specific guidelines should be lauded, but our data suggests that methods to enforce the guidelines may also be needed.

## Supporting information

S1 Table Twenty-one item reporting checklist developed from the deconstructed and operationalized NIH-PRG. Note that questions are not ordered by domain.
(PDF)

S2 Table Country of residency of the corresponding author at the time of article publication. (PDF)

S3 Table Reported sources of funding. The total number of sources was 844 as a study can be funded by more than one body.
(PDF)

S4 Table Broad topic of investigation based on International Statistical Classification of Diseases and Related Health Problems (ICD-10). (PDF)

S5 Table Animal model characteristics. Species and number of animal used in preclinical anesthesiology studies. Note that some studies used more than one species for a total of 617 animal models.
(PDF)

S6 Table Results from the assessment on preclinical *in vivo* study design. Level of reporting across all included studies (N = 604) against the deconstructed National Institutes of Health preclinical reporting guidelines (NIH-PRG). These recommendations are grouped into seven domains, from which 21 unidimensional items were identified and operationalized into ‘yes’ or ‘no’ questions. The number of times each item was reported is displayed as n (%).
(PDF)

S7 Table The effect of the listed use of reporting guidelines. Comparative analysis of the level of reporting in articles which used reporting guidelines compared to those that did not against the NIH-PRG; note that 26 listed the ARRIVE guidelines and 1 listed Utstein-Style guidelines. For clarity the descriptions have been shortened.
(PDF)

S8 Table The effect of endorsing reporting guidelines. Comparison of completeness of reporting between journals that endorsed preclinical reporting guidelines *(British Journal of Anaesthesia*, *Anaesthesia)* and those that did not (*Anesthesiology* and *Anesthesia & Analgesia*) against the NIH-PRG. For clarity the descriptions have been shortened.
(PDF)

S9 Table The effect of the publication of reporting guidelines. Comparison of completeness of reporting over time against the NIH-PRG; pre-ARRIVE (2008, 2009) versus post-ARRIVE (2014–2016) publications. For clarity, the descriptions have been shortened.
(PDF)
