# Supplementary material for: Reporting preclinical anesthesia study (REPEAT): Evaluating the quality of reporting in the preclinical anesthesiology literature
Source: PLoS One. 2019 May 23;14(5):e0215221. doi: 10.1371/journal.pone.0215221 (PMC6532843; doi:10.1371/journal.pone.0215221)
Supplement: S1 Table — Note that questions are not ordered by domain. (PDF) [file pone.0215221.s001.pdf]

Question	Yes	No
**Were reporting guidelines listed in the study?**<br>Note<br>This refers to community based standards;<br>e.g. ARRIVE guidelines, Gold Standard Publication Checklist, etc.		
**Were any of the results substantiated by repetition under a range of conditions in the study?**<br>Note<br>These include (but are not limited to) independent experiments with different drug dosage, duration, and types of intervention;<br>e.g. State 4 respiration, however, was affected by ketamine administration, increasing for K50 (P=0.023), K100 (P=0.012), and K150 (P=0.015) versus the K0 group.		
**Did the study list statistical tests used in the methods?**<br>e.g. T-test, ANOVA, etc.		
**Did the study explicitly state the total number of animals used?**<br>Note<br>Addition of number of animals in each experimental group should not be necessary.		
**Did the study state the exact value of N for all in vivo experiments?**<br>Note<br>N represents the number of animals per group.		
**Was the measure of central tendency stated?**<br>e.g. Group data are presented as means±SD for infarct volumes.•		
**Did the study state the measure of dispersion?**<br>Note<br>Measures of dispersion include (but not limited to) standard deviation, standard error and confidence intervals.		
**Were samples randomly divided into each group?**<br>Note<br>The word randomized is used;<br>e.g. ...mice were randomly allocated to 5 groups.		
**Did the study describe the method of randomization?**<br>e.g. Random sequence generator was used to allocate animals.		
**Were the experimenters and/or caregivers blinded when conducting experiments?**<br>e.g. The person conducting behaviour was blinded to treatment group.		
**Were the personnel analyzing results blinded to outcome assessments?**<br>e.g. Behavioural tests were carried out 24h postocclusion by an investigator blinded to treatment allocation.		
**Was the primary outcome explicitly stated?**		
**Did the study indicate whether the sample size was calculated?**<br>e.g. A sample size calculation was used to determine the number of animals needed to be used in experiment.		
**Did the study include the statistical method of sample size calculation?**<br>Note<br>Method of sample size calculation includes (but not limited to) resource equation method, sample size calculation method, etc.;<br>e.g. Sample size calculations were performed in STATA 10 with the sampsi function.		

**<u>Does the study state the criteria used to exclude any data/subjects/results from analysis or does the study state that some samples were excluded for any reason or does the study explicitly state that there were no exclusions of data/subjects/results?</u>**<br>Note<br>These include (but are not limited to) eligibility criteria;<br>e.g. The animals were excluded if insertion of the thread resulted in perforation of the vessel wall (determined by the presence of sub-arachnoid blood at the time of sacrifice.		
**<u>Were there experiments performed/listed in the methods section that were not reported in the results section?</u>**<br>Note<br>This refers to selective reporting bias.		
**<u>Were any pilot or preliminary studies reported?</u>**<br>Please note for this question it does not matter if the pilot/preliminary results were negative.		
**<u>Were there any conditions reported/measured that led to negative results?</u>**<br>Note<br>For this question negative results need to be explicitly reported (in text, figures, tables, etc.).<br>Negative results pertain to results that do not support the main findings not hypothesis.<br>These include (but are not limited to) pilot studies that showed negative results, p-values that were not significant, qualitative results that are contradictory, and first few doses not having an effect.		
**<u>For at least one experimental outcome, are the number of subjects (n) per group explicitly stated?</u>**		
**<u>For at least one experimental outcome, are the number of measurements per subject explicitly stated?</u>**		
**<u>Were the above criteria met for all experimental outcomes?</u>**		
