# Supplementary material for: Reporting preclinical anesthesia study (REPEAT): Evaluating the quality of reporting in the preclinical anesthesiology literature
Source: PLoS One. 2019 May 23;14(5):e0215221. doi: 10.1371/journal.pone.0215221 (PMC6532843; doi:10.1371/journal.pone.0215221)
Supplement: S2 Table — (PDF) [file pone.0215221.s002.pdf]

Question	Country	Frequency, n
Indicate corresponding author's country of residence.	United States	216
	China	66
	Japan	62
	Germany	53
	Canada	26
	France	24
	Taiwan	17
	Netherlands	15
	United Kingdom	13
	Spain	12
	Brazil	11
	Sweden	10
	South Korea	9
	Australia	9
	Italy	8
	Switzerland	7
	New Zealand	6
	Belgium	5
	Turkey	5
	Austria	4
	Serbia	4
	Denmark	3
	Finland	3
	Ireland	3
	Israel	3
	Norway	2
	Portugal	2
	Slovenia	2
	Croatia	1
	Hungary	1
	India	1
	Iran	1
