# Supplementary material for: Reporting preclinical anesthesia study (REPEAT): Evaluating the quality of reporting in the preclinical anesthesiology literature
Source: PLoS One. 2019 May 23;14(5):e0215221. doi: 10.1371/journal.pone.0215221 (PMC6532843; doi:10.1371/journal.pone.0215221)
Supplement: S3 Table — The total number of sources was 844 as a study can be funded by more than one body. (PDF) [file pone.0215221.s003.pdf]

| Question | Response | Frequency, n |
|---|---|---|
| Indicate the source of any research funding. | Government | 416 |
| | Academic | 203 |
| | Industry | 77 |
| | Explicitly unfunded | 2 |
| | No mention of funding | 37 |
| | Other | 109 |
