# Supplementary material for: Reporting preclinical anesthesia study (REPEAT): Evaluating the quality of reporting in the preclinical anesthesiology literature
Source: PLoS One. 2019 May 23;14(5):e0215221. doi: 10.1371/journal.pone.0215221 (PMC6532843; doi:10.1371/journal.pone.0215221)
Supplement: S4 Table — (PDF) [file pone.0215221.s004.pdf]

| Question | Response | Frequency, n |
|---|---|---|
| What was the broad topic investigated in this study? | Pain and analgesia | 180 |
| | Critical illness | 77 |
| | Cardiovascular system | 75 |
| | Nervous system | 70 |
| | Toxicology | 54 |
| | Drug pharmacokinetics and/or pharmacodynamics | 48 |
| | Respiratory system | 42 |
| | Device/instrument evaluation | 23 |
| | Blood and blood forming organs, immune mechanism/disorder | 9 |
| | Musculoskeletal system and connective tissue | 4 |
| | Mental and behavioral disorders | 4 |
| | Other | 4 |
| | Cancer | 2 |
| | Digestive System | 2 |
| | Genitourinary System | 2 |
| | Skin and subcutaneous tissue | 2 |
| | Endocrine, nutritional, and metabolic disease | 2 |
| | Certain conditions originating in the perinatal period | 2 |
| | Eye | 2 |
