# Supplementary material for: Reporting preclinical anesthesia study (REPEAT): Evaluating the quality of reporting in the preclinical anesthesiology literature
Source: PLoS One. 2019 May 23;14(5):e0215221. doi: 10.1371/journal.pone.0215221 (PMC6532843; doi:10.1371/journal.pone.0215221)
Supplement: S5 Table — Species and number of animal used in preclinical anesthesiology studies. Note that some studies used more than one species for a total of 617 animal models. (PDF) [file pone.0215221.s005.pdf]

Response	Number of Studies	Number of Subjects Used
Rat	338	32223
Mouse	132	8983
Rabbit	34	3026
Pig / Swine	75	2448
Guinea Pig	3	522
Dog	15	478
Frog	3	278
Ewes / Sheep	9	246
Cat	3	178
Non-human Primate	3	45
Goat	1	30
Fruit Fly	1	4033
