# Supplementary material for: Reporting preclinical anesthesia study (REPEAT): Evaluating the quality of reporting in the preclinical anesthesiology literature
Source: PLoS One. 2019 May 23;14(5):e0215221. doi: 10.1371/journal.pone.0215221 (PMC6532843; doi:10.1371/journal.pone.0215221)
Supplement: S6 Table — Level of reporting across all included studies (N = 604) against the deconstructed National Institutes of Health preclinical reporting guidelines (NIH-PRG). These recommendations are grouped into seven domains, from which 21 unidimensional items were identified and operationalized into ‘yes’ or ‘no’ questions. The number of times each item was reported is displayed as n (%). (PDF) [file pone.0215221.s006.pdf]

Domain	Item Description	n (%) reported **(N=604)**
Standards	Community based reporting guidelines listed	27 (4)
Replicates (biological vs. technical)	Results substantiated by repetition under a range of conditions	574 (95)
	Number of subjects per outcome	477 (79)
	Number of measurements per subject for one experimental outcome stated	108 (18)
	Number of measurements per subject for all experimental outcomes stated	2 (0.3)
Statistics	List of the total number of subjects used in each experimental group	501 (83)
	List of all statistical tests used	597 (99)
	Definition of the measure of central tendency	597 (99)
	Definition of the measure of dispersion and precision	597 (99)
Randomization	Random group assignment reported	319 (53)
	Description of the method of random group assignment	63 (10)
Blinding	Experimenters blinded to group allocation during conduct of the experiment	113 (19)
	Experimenters blinded to group allocation during result assessment	245 (41)
Sample Size Estimation	Description of an *a priori* primary outcome	49 (8)
	Sample size computed during study design	136 (23)
	Description of the method of sample size determination	76 (13)
Inclusion and Exclusion Criteria	Total number of animals procured for the experiment reported	273 (45)
	Description of the criteria used for the exclusion of any data or subjects	198 (33)
	Description of any outcomes that were measured and not reported in the results section	593 (98)
	Pilot or preliminary studies performed and listed	192 (32)
	Null or negative outcomes included in the results	539 (89)
