# Supplementary material for: Reporting preclinical anesthesia study (REPEAT): Evaluating the quality of reporting in the preclinical anesthesiology literature
Source: PLoS One. 2019 May 23;14(5):e0215221. doi: 10.1371/journal.pone.0215221 (PMC6532843; doi:10.1371/journal.pone.0215221)
Supplement: S7 Table — Comparative analysis of the level of reporting in articles which used reporting guidelines compared to those that did not against the NIH-PRG; note that 26 listed the ARRIVE guidelines and 1 listed Utstein-Style guidelines. For clarity the descriptions have been shortened. (PDF) [file pone.0215221.s007.pdf]

Domain	Description	n (%) reported<br>No Guidelines N=577	n (%) reported<br>Guidelines N=27	RR (95% CI)
Standards	Reporting guidelines	0 (0)	27 (100)	N/A
Replicates	Range of conditions	519 (90)	22 (81)	0.91 (0.76-1.09)
	Number of subjects per outcome	456 (79)	23 (85)	1.08 (0.92-1.27)
	Number of measurements per outcome	104 (18)	5 (19)	1.03 (0.46-2.31)
	Number of measurements per subject per outcome	0 (0)	0 (0)	N/A
Statistics	Total number of subjects	473 (82)	25 (93)	1.13 (1.01-1.27)
	Statistical tests used	571 (99)	27 (100)	0.99 (0.94-1.05)
	Measure of central tendency	571 (99)	27 (100)	0.99 (0.94-1.05)
	Measure of dispersion	571 (99)	27 (100)	0.99 (0.94-1.05)
Randomization	Random group assignment	317 (55)	22 (81)	1.48 (1.22-1.80)
	Method of randomization	104 (18)	13 (47)	2.67 (1.74-4.10)
Blinding	Group allocation blinding	110 (19)	6 (22)	1.17 (0.56-2.41)
	Result assessment blinding	237 (41)	12 (44)	1.08 (0.70-1.67)
Sample Size Estimation	Primary outcome	46 (8.0)	3 (11)	1.39 (0.46-4.20)
	Sample size calculation	127 (22)	9 (33)	1.51 (0.97-2.64)
	Mathematical method used	323 (56)	15 (56)	0.99 (0.70-1.40)
Inclusion and Exclusion Criteria	Total number of animals	260 (45)	13 (48)	1.07 (0.72-1.60)
	Data/subjects/results exclusion	185 (32)	11 (41)	1.27 (0.79-2.03)
	No result omissions	565 (98)	27 (100)	1.00 (0.95-1.06)
	Pilot/preliminary studies	185 (32)	9 (33)	1.04 (0.60-1.80)
	Null/negative results	519 (90)	22 (81)	0.91 (0.76-1.09)
