# Supplementary material for: Reporting preclinical anesthesia study (REPEAT): Evaluating the quality of reporting in the preclinical anesthesiology literature
Source: PLoS One. 2019 May 23;14(5):e0215221. doi: 10.1371/journal.pone.0215221 (PMC6532843; doi:10.1371/journal.pone.0215221)
Supplement: S8 Table — Comparison of completeness of reporting between journals that endorsed preclinical reporting guidelines (British Journal of Anaesthesia, Anaesthesia) and those that did not (Anesthesiology and Anesthesia & Analgesia) against the NIH-PRG. For clarity the descriptions have been shortened. (PDF) [file pone.0215221.s008.pdf]

Domain	Description	n (%) reported Endorsed (N=41)	n (%) reported Unendorsed (N=241)	RR (95% CI)
Standards	Reporting guidelines	24 (59)	2 (0.8)	70.54 (17.33-287.18)
Replicates	Range of conditions	33 (80)	228 (95)	0.85 (0.73-0.99)
	Number of subjects per outcome	33 (80)	201 (83)	0.97 (0.82-1.13)
	Number of measurements per outcome	4 (10)	45 (19)	0.52 (0.20-1.37)
	Number of measurements per subject per outcome	0 (0)	1 (0.4)	1.92 (0.08-46.36)
Statistics	Total number of subjects	35 (85)	207 (86)	0.99 (0.87-1.14)
	Statistical tests used	39 (95)	240 (100)	0.96 (0.89-1.02)
	Measure of central tendency	41 (100)	239 (99)	1.00 (0.96-1.03)
	Measure of dispersion	40 (98)	239 (99)	0.98 (0.94-1.03)
Randomization	Random group assignment	24 (59)	140 (58)	1.01 (0.76-1.33)
	Method of randomization	13 (32)	26 (11)	2.94 (1.65-5.24)
Blinding	Group allocation blinding	4 (10)	56 (23)	0.42 (0.16-1.10)
	Result assessment blinding	15 (37)	113 (47)	0.78 (0.51-1.19)
Sample Size Estimation	Primary outcome	6 (15)	26 (11)	1.36 (0.60-3.09)
	Sample size calculation	11 (27)	72 (30)	0.90 (0.52-1.54)
	Method used for calculation	8 (20)	46 (19)	1.02 (0.52-2.01)
Inclusion and Exclusion Criteria	Total number of animals	25 (61)	86 (36)	1.71 (1.27-2.30)
	Data/subjects/results exclusion	19 (46)	84 (35)	1.33 (0.92-1.93)
	No result omissions	0 (0)	152 (63)	0.02 (0.0-0.30)
	Pilot/preliminary studies	12 (29)	83 (34)	0.85 (0.51-1.41)
	Null/negative results	33 (80)	219 (91)	0.89 (0.76-1.04)
