# Supplementary material for: Reporting preclinical anesthesia study (REPEAT): Evaluating the quality of reporting in the preclinical anesthesiology literature
Source: PLoS One. 2019 May 23;14(5):e0215221. doi: 10.1371/journal.pone.0215221 (PMC6532843; doi:10.1371/journal.pone.0215221)
Supplement: S9 Table — Comparison of completeness of reporting over time against the NIH-PRG; pre-ARRIVE (2008, 2009) versus post-ARRIVE (2014–2016) publications. For clarity, the descriptions have been shortened. (PDF) [file pone.0215221.s009.pdf]

Domain	Description	n (%) reported 2008, 2009 N=322	n (%) reported 2014-2016 N=282	RR (95% CI)
Standards	Reporting guidelines	N/A	26 (9)	N/A
Replicates	Range of conditions	312 (97)	262 (93)	0.96 (0.92-0.99)
	Number of subjects per outcome	242 (75)	234 (83)	1.10 (1.02-1.20)
	Number of measurements per outcome	58 (18)	48 (17)	0.95 (0.67-1.34)
	Number of measurements per subject per outcome	0 (0)	0 (0)	N/A
Statistics	Total number of subjects	258 (80)	243 (86)	1.08 (1.01-1.16)
	Statistical tests used	319 (99)	279 (99)	1.00 (0.98-1.02)
	Measure of central tendency	316 (98)	279 (99)	1.01 (0.99-1.03)
	Measure of dispersion	319 (99)	279 (99)	1.00 (0.98-1.02)
Randomization	Random group assignment	161 (50)	178 (63)	1.26 (1.10-1.45)
	Method of randomization	48 (15)	68 (24)	1.62 (1.16-2.26)
Blinding	Group allocation blinding	52 (16)	59 (21)	1.30 (0.93-1.81)
	Result assessment blinding	119 (37)	127 (45)	1.22 (1.01-1.48)
Sample Size Estimation	Primary outcome	16 (5.0)	31 (11)	2.21 (1.24-3.96)
	Sample size calculation	52 (16)	82 (29)	1.80 (1.32-2.45)
	Method used for calculation	135 (42)	183 (65)	1.55 (1.33-1.81)
Inclusion and Exclusion Criteria	Total number of animals	161 (50)	110 (39)	0.78 (0.65-0.94)
	Data/subjects/results exclusion	97 (30)	104 (37)	1.22 (0.98-1.53)
	No result omissions	312 (97)	282 (100)	1.03 (1.01-1.05)
	Pilot/preliminary studies	97 (30)	96 (34)	1.13 (0.90-1.43)
	Null/negative results	287 (89)	251 (89)	1.00 (0.94-1.06)
